# The Two Bills in Congress

**Published:** 1894-03

**Authors:** 


### THE TWO BILLS IN CONGRESS.

It is an unfortunate thing that the medical profession cannot agree upon what legislation is needed in the interest of the public health. As is well known, there is no official to whom Congress can apply for the necessary data upon which to frame sanitary laws,—as can be done with regard to legislation upon every other interest under the sun. Should data be needed upon agriculture, cattle-raising, finance, war, labor, immigration, shlpping,—anything, there is a source where it is at once obtainable; not so as to sanitation; and in the absence of such official it would be a difficult matter to determine what is needed. This is the predicament Congress is in; they do not know what to enact.

We have on hand in the matter, really, amongst medical men, a three-handed fight. The friends of the Marine Hospital Service insist that the law of February, 1893 (which is only a quarantine law) is good enough, and oppose any change. A majority of the N. Y. Academy of Medicine have a bill before Congress which proposes to take the administration of quarantine out of the hands of the Marine Hospital Service and put it in the hands of a commission of fifteen doctors. Their bill, as one of the minority members said, is no improvement on the one now in force; it provides for nothing which is not now provided for, and which is being better and less clumsily done under the law now in force.

Both these parties seem to think that quarantine is all of sanitation. They are, moreover, opposed to State quarantine, and no doubt would be glad to see the National government take quarantine out of the hands of the State governments altogether. And, by the bye, Dr. Cochran, at the late Sanitary Conference in New Orleans, predicted that he would live to see the day when it will be done. God forbid.

The American Medical Association, on the other hand, is a representative body—the only one in existence—of the profession of the whole country, and are not biased by any local prejudices. They take a more comprehensive view of sanitation. To paraphrase a beautiful sentiment—they realize that "it is not all of sanitation to establish quarantine;" they would prevent disease. They ask for the passage of a bill that provides for something besides maritime quarantine; they ask that a department of public health be created, in the first place, with an executive officer at the head of it, thus putting the great interests of public health on a footing with all other interests. Such an officer would then be the proper person to know, and would know, and would be able to advise the law makers what is needed in the way of sanitary legislation. Congressmen would then not be embarrassed, as they now are, by conflicting opinions amongst medical men. Congress means well, there is no doubt; we have had every evidence of it. Members would be glad to make such laws as will insure the safety of life, and put the whole country on the highest plane of sanitary protection, if they could find out what enactments will do it; they do not know, the doctors do not agree, and there is no way to find out.

It would seem the most natural and the most sensible thing to do, under the circumstances, to be guided by advice emanating from the American Medical Association. Its voice comes nearer expressing the wishes of the medical profession than that of any local organization, or any interested party; and as the membership represents the entire medical profession of America, who, being in closer touch with the people in this relation than any other class, are in position to know better than any local organization what kind of legislation will best serve to bring America up to the plane occupied by other nations in sanitation. It is a humiliating thing to be told, as we were recently, by cable dispatches, that American sanitation is the laughing stock of Europe.

We give herewith the text of the bill to create a Public Health Department, that our readers may have an idea of the real requirements. The bill of the New York Academy is almost a duplicate of the Quarantine law passed February, 1893; the only difference being the manner of administering quarantine. The

Sanitarians all over the country, notably in Chicago and Memphis, have condemned the bill; and we opine it will be pigeonholed to see daylight never more.

Every medical man who feels any interest in the wellfare of his country, or has any pride in his profession, should use what influence lies in his power to aid in the passage of the American Medical Association bill. A letter to a Congressman may open his eyes to facts he had not even dreamed of, and may influence his vote upon this important question.

